# X-Ray Computed Tomography Instrument Performance Evaluation, Part III: Sensitivity to Detector Geometry and Rotation Stage Errors at Different Magnifications

**DOI:** 10.6028/jres.126.029

**Published:** 2021-09-29

**Authors:** Prashanth Jaganmohan, Bala Muralikrishnan, Meghan Shilling, Edward Morse

**Affiliations:** 1 Sensor Science Division, National Institute of Standards and Technology, Gaithersburg, MD 20899, USA; 2 Center for Precision Metrology, University of North Carolina at Charlotte, Charlotte, NC 28223, USA

**Keywords:** cone-beam, distance error, documentary standards, form error, geometry errors, performance evaluation, radiograph-based method, sensitivity analysis, single-point ray tracing method, X-ray computed tomography

## Abstract

With steadily increasing use in dimensional metrology applications, especially for delicate parts and those with complex internal features, X-ray computed tomography (XCT) has transitioned from a medical imaging tool to an inspection tool in industrial metrology. This has resulted in the demand for standardized test procedures and performance evaluation standards to enable reliable comparison of different instruments and support claims of metrological traceability. To meet these emerging needs, the American Society of Mechanical Engineers (ASME) recently released the B89.4.23 standard for performance evaluation of XCT systems. There are also ongoing efforts within the International Organization for Standardization (ISO) to develop performance evaluation documentary standards that would allow users to compare measurement performance across instruments and verify manufacturer’s performance specifications. Designing these documentary standards involves identifying test procedures that are sensitive to known error sources. This paper, which is the third in a series, focuses on geometric errors associated with the detector and rotation stage of XCT instruments. Part I recommended positions of spheres in the measurement volume such that the sphere center-to-center distance error and sphere form errors are sensitive to the detector geometry errors. Part II reported similar studies on the errors associated with the rotation stage. The studies in Parts I and II only considered one position of the rotation stage and detector; i.e., the studies were conducted for a fixed measurement volume. Here, we extend these studies to include varying positions of the detector and rotation stage to study the effect of magnification. We report on the optimal placement of the stage and detector that can bring about the highest sensitivity to each error.

## 1 Introduction

The use of X-ray computed tomography (XCT) in industrial metrology has been steadily increasing in recent years as a means for dimensional inspection [1–2]. This is especially true for complex parts where traditional inspection procedures would prove time-consuming or impossible. For example, fragile parts or parts with internal features such as those manufactured through additive manufacturing cannot be probed or accessed by contact-based measurement methods or line-of-sight optical methods. These distinct advantages in XCT have brought about the transition from its predominant use in defect inspection and medical imaging into dimensional measurements for engineering applications [3]. As a result of such increasing demand and widespread use, there is a need for standardized test procedures to evaluate XCT instrument performance and to support claims of metrological traceability. This need was articulated in several publications in the early and mid-2000s [4–6].

To meet this need, the International Organization for Standardization (ISO) and the American Society of Mechanical Engineers (ASME) began working independently to develop XCT performance evaluation standards [7–8]. ASME recently completed their work, resulting in the publication of the B89.4.23 standard in early 2021 [8]. There are also published guidelines from the Association of German Engineers and the Association of German Electrical Engineers, the VDI/VDE, for specifying and testing the accuracy of industrial XCT systems [9–11]. Note that there are other documentary standards published by ASTM International [12–13] and ISO [14], but they address imaging issues and are primarily intended for nondestructive evaluation, not dimensional measurements. There is currently ongoing discussion within ASTM to develop a documentary standard for dimensional inspection, but those discussions are at a very early stage.

In the writing of performance evaluation standards, one of the primary goals is to design test procedures that are sensitive to as many known error sources as possible. The first step is to identify all possible error sources and understand the effect of each individual error source on dimensional measurements. VDI/VDE 2630-1.2 [10] lists and discusses several error sources associated with XCT systems. We have described the influence of uncorrected instrument geometry errors in cone-beam XCT instruments in a series of papers, of which this is the third part. In Part I of this series [15], we described a new simulation method, referred to as the single-point ray tracing method (SPRT), that enables rapid simulation of the effect of instrument geometry errors without the need for generating radiographs and performing tomographic reconstruction. We then utilized this technique to describe the effect of detector geometry errors on the sphere center-to-center distance error and sphere form error. In Part II of this series [16], we focused on the effect of rotation stage errors on the sphere center-to-center distance error and sphere form error, again using SPRT. Those studies identified the placement of spheres in the measurement volume so that each of the error sources could be captured most effectively, *i.e.*, at their maximum sensitivities. Those studies also provided recommendations for documentary standards committees to consider as they developed performance evaluation procedures. However, those studies were limited to a fixed measurement volume, *i.e.*, for chosen fixed positions of the detector (1177 mm from source) and the rotation stage (400 mm from the source) based on prior work reported by Ferrucci *et al*. [17].

Many XCT systems allow both the detector and the rotation stage to move, enabling numerous imaging magnifications. In fact, the same magnification can sometimes be realized through different combinations of source-stage (*d*) and source-detector (*D*) distances. To capture the effect of magnification, the ASME B89.4.23 standard and current draft of the ISO standard advocate testing at different measurement volumes (which can be realized by changing the positions of the rotation stage and/or detector), but they do not provide comprehensive guidance as to how the user should select these measurement volumes or all of the measurement lines within these volumes. Thus, we identified the need to extend the studies in Parts I and II to cover the working range of an XCT system with a movable detector so that we may provide more specific guidance to documentary standards committees developing these documents and to users of such systems that want to establish whether their instrument meets the manufacturer’s specifications. In this Part III, we extend the work done in the first two parts [15–16] by repeating the SPRT simulations for several combinations of *d* and *D*. We discussed some early results in Ref. [18]; here, we present a more comprehensive description of the results and conclusions.

First, we briefly review literature in the area of performance evaluation of XCT systems using calibrated reference objects, particularly using spheres as metrological elements, since that is the method adopted by documentary standards committees. The use of a calibrated reference object consisting of ruby spheres mounted on shafts has been used reported by a number of researchers [4, 19–21]. This reference object, often referred to as a sphere forest, is currently under consideration by ISO as a reference object for evaluating the performance of XCT systems. The use of calibrated reference objects such as a ball-bar [22], a tetrahedron of spheres [23, 24], a ball-plate or a hole-plate [25–27], and spheres on a cylinder [28] has also been reported for performance evaluation of XCT systems. A summary of different reference objects used for evaluating performance of XCT systems can be found in Ref. [29]. The effect of XCT geometry errors on cone-beam XCT systems has been discussed in Refs. [30–32]. For a more general review of related literature on geometry errors in XCT systems, see Ferrucci *et al*. [33].

The rest of this paper is organized as follows. We describe the reference object used in the simulations and the different geometric error sources in Sec. 2. We discuss the simulation technique employed in Sec. 3. We present our results in Sec. 4 and conclusions in Sec. 5.

## 2 Reference Object and Error Sources

**Fig. 1:**
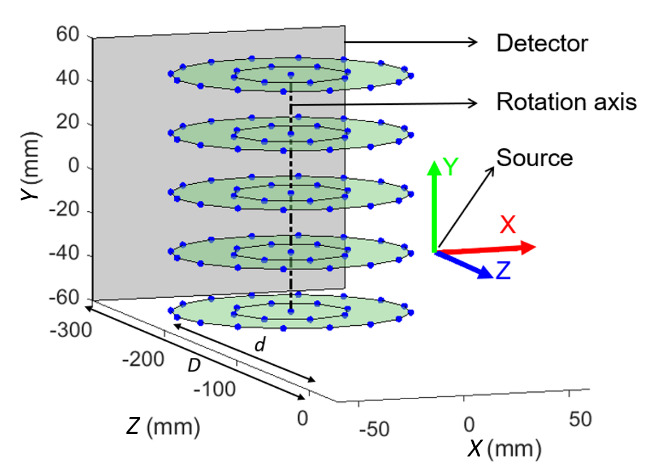
Reference object containing 125 spheres chosen for simulation.

All simulations in the present work were carried out using a simulated reference object consisting of 125 spheres evenly distributed into five horizontal planes, each containing one centrally located sphere, 16 spheres arranged in an outer circle, and 8 spheres arranged in a smaller circle of half the diameter of the outer circle, as shown in Fig. 1. This is the same sphere arrangement described in Ref. [15]. In Fig. 1, the source and detector positions are drawn to scale, but the detector size is not. The coordinate system used in this model has its origin at the source, and it has its axes directed as shown in Fig. 1. Detailed descriptions on establishing the coordinate system by defining each axis can be found in Ref. [17]. While previous studies [15–16] considered the object to be of a single fixed size, in this study the height and diameter of the object’s cylindrical shape are functions of *d* and *D*. In other words, the simulated object is scaled for each combination of source-stage and source-detector distance so that 98% of the area of a 250 mm × 250 mm continuous (*i.e.*, nonpixelated) detector is filled. This scaling is done while ensuring that all the spheres on the boundaries of the reference object are fully within the detector field of view (FOV). The purpose of such scaling is to obtain the largest possible magnitudes of distance errors for each combination of stage and detector positions. The sphere diameters are also scaled accordingly while ensuring that their projected images are within the detector FOV.

The geometric error sources associated with the detector and stage are described in detail in Parts I and II [15–16] and in Ferrucci *et al*. [32–33]. We present a brief summary here in the interest of completeness. The detector errors include the three location errors along each Cartesian axis of the instrument coordinate frame, and three angular positioning errors about the same axes. These are described in Table 1, where the assumed magnitudes of geometric errors are also included.

**Table 1:** Description of detector errors.

Symbol/ Error Source	Description	Assumed Magnitude
$e_{x}$	Detector location error along the *X* axis	1.27 mm
$e_{y}$	Detector location error along the *Y* axis	1.27 mm
$e_{z}$	Detector location error along the *Z* axis	0.1 mm
$\theta_{x}$	Detector rotation error about an axis parallel to *X* axis	0.2°
$\theta_{y}$	Detector rotation error about an axis parallel to *Y* axis	1°
$\theta_{z}$	Detector rotation error about an axis parallel to *Z* axis	0.2°

The stage errors include a *Z* location error (along the axis connecting the source and rotation axis), and the error motions of the stage itself. These error motions, including encoder scale, axial, radial, and wobble errors (often referred to as tilt errors in documentary standards), are all assumed to have harmonic components and therefore are represented as sine and cosine functions of the rotation stage indexing angle, of the form $a\sin\left(n\theta \right)$ and $a\cos\left(n\theta \right)$, where *a* represents the amplitude or magnitude of the error, *n* is the order of the harmonic, and *θ* is the table indexing angle. This study included harmonics of orders one through ten. These stage errors are described in Table 2, where the corresponding magnitudes are also included. In the case of the error motions, the magnitudes shown in Table 2 refer to the amplitude *a*. The complete value of the geometric error in these cases will depend on *a*, *n*, and *θ* as described above. The *X* and *Y* positioning errors of the stage have no physical meaning due to the way the coordinate system is defined [15].

**Table 2:** Description of stage errors.

Symbol/ Error Source	Description	Assumed Magnitude
$d_{z}$	Error in the location of the rotation axis along *Z* axis	0.1 mm
$e_{ax}$	Axial error motion (along *Y* axis)	0.1 mm (amplitude)
$e_{r,x}$	Radial error motion component along *X* axis	0.1 mm (amplitude)
$e_{r,z}$	Radial error motion component along *Z* axis	0.1 mm (amplitude)
$e_{w,x}$	*X* component of the wobble error	0.2° (amplitude)
$e_{w,z}$	*Z* component of the wobble error	0.2° (amplitude)
$e_{\theta }$	Error in the angular position of the stage (*i.e.*, encoder scale error)	0.05° (amplitude)

For any given geometry error parameter described in Tables 1 and 2, we performed (but not reported here) simulations for different magnitudes of the introduced geometry error to ensure that the center-to-center distance error and sphere form error do in fact have a linear relationship with the introduced geometry error.

## 3 Methodology

The SPRT method introduced and described in detail in Ref. [15] is the simulation method adopted in this work. This method has been experimentally validated and has proven to be a faster and more practical alternative to the full XCT tomographic reconstruction methods for the purposes of estimating the effects of geometric errors using sphere-based objects. In the SPRT method, only the sphere centers are projected onto the detector, as opposed to the more traditional radiograph-based reconstruction of the entire object. As the stage makes a full rotation, the projection of the center of each sphere on the detector traces a locus. These obtained loci are used to determine the location of each sphere in the measurement volume through a least-squares-minimization–based back-projection algorithm. From the determined sphere centers, the center-to-center distances for each pair of the 125 sphere centers are determined. To estimate form error, circles consisting of 120 equally spaced points are constructed normal to each ray connecting the source and the detector, with their centers located on the previously identified least-squares centers. This is performed for each angular position of the stage as the stage rotates, and therefore the circles at different rotations form the spherical surface [15]. The diameters of these circles correspond to the scaled diameters of the spheres in the reference object. The points lying in the interior of a convex hull generated from the resulting point cloud are truncated, and only the outer points are used for form error calculation. Here, we define form error as the difference between the maximum and minimum residuals from a least-squares best-fit sphere to the point-cloud data. While such a peak-to-valley approach is generally sensitive to outliers, the likelihood of outliers from the SPRT is small to none, due to its concept.

When a particular error source is analyzed, the assumed magnitude of the geometric error is used in the forward projection. For example, the actual value of $\theta_{y}$ is used to generate the simulated locus for each sphere. However, in the back-projection algorithm, an errorless instrument is assumed. This discrepancy between actual and assumed geometry parameters represents the magnitude of simulated errors and results in sphere center-to-center distance errors and sphere form errors. In this way, the effects of all geometry errors associated with the detector and stage on the center-to-center distance errors and form errors of the spheres on the reference object are studied. In this work, such simulations were performed at several stage and detector positions to study the effect of magnification.

For each error source under consideration, the pair of spheres that produced the highest center-to-center distance error was previously identified in Parts I and II for a specific combination of *d* and *D*. The line joining this pair of spheres constitutes the line of highest sensitivity, *i.e.*, center-to-center distance error (in mm) per millimeter or degree of geometric error. In this study, we tracked this pair of spheres across all stage and detector positions to identify the specific combination of *d* and *D* that resulted in the largest distance error. Similarly, the sphere producing the highest form error was previously identified in Parts I and II for one combination of *d* and *D*. In this study, this sphere was tracked through all combinations of stage and detector positions to identify the specific combination of *d* and *D* that resulted in the largest form error. The pairs of spheres producing the highest distance error as identified in Parts I and II, or the spheres producing the highest form error also identified therein, may not always be the same at all combinations of *d* and *D*.

**Fig. 2:**
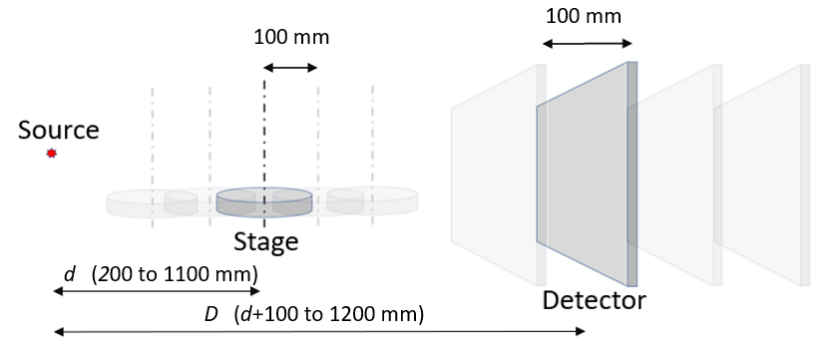
Schematic showing the different rotation stage and detector positions considered in the simulation.

In this study, we considered a detector that can travel up to a distance of 1200 mm from the source. In the simulations, *d* was varied from 200 mm to 1100 mm in steps of 100 mm. For each position of the stage, *D* was varied from *d* + 100 mm to 1200 mm, in steps of 100 mm. The diameter of the spheres in the appropriately scaled reference object ranged from 3.40 mm to 18.68 mm at the smallest and largest measurement volume, respectively. Figure 2 shows an overview of this approach.

## 4 Results and Discussion

In this paper, we have intentionally chosen a different approach for the presentation of the results from the previous parts. In Parts I and II, we discussed each error source separately, highlighting the spheres that produced the maximum distance error and the sphere that produced the largest form error. The results in those studies were based on just one simulation (for the specific case of *d* and *D* considered there) for each of the six detector errors and 121 rotation stage errors. Thus, in total, 127 simulations were performed. In this study, we perform 55 simulations (corresponding to all combinations of *d* and *D* considered here) for each of the six detector errors and the 121 rotation stage errors, resulting in a total of 6985 simulations. Given the enormous number of simulations, we intentionally chose to distill the information and present key findings instead of discussing each error source independently, as we did in Parts I and II.

### 4.1 Sphere Center-to-Center Distance Errors

#### 4.1.1 Detector Errors

**Fig. 3:**
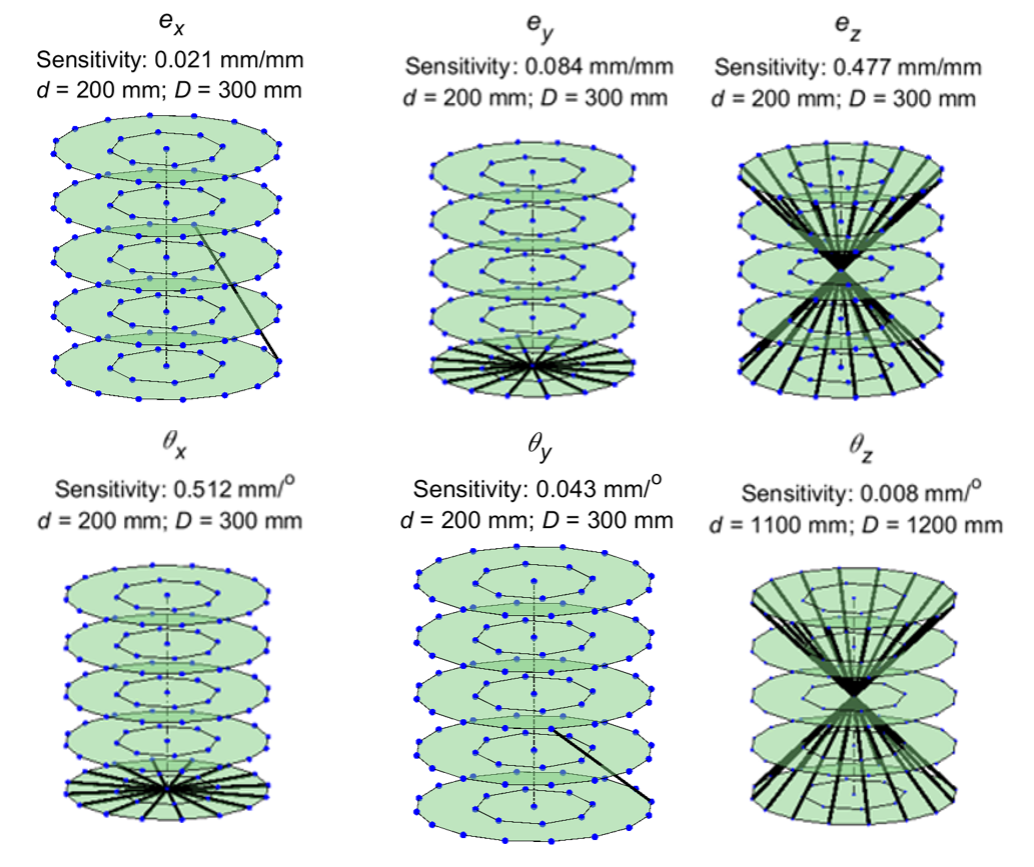
Lines of maximum sensitivity for detector errors.

The lines of maximum sensitivity for each of the six error sources associated with the detector are shown in Fig. 3. Cases where multiple lines are shown for a single error source indicate that all of the lines shown are equally sensitive. The values of *d* and *D* for which this highest sensitivity is observed are also mentioned in each case.

For each error source, we plotted the distance error of the corresponding sensitive center-to-center length segment from Part I as a function of *d* and *D*. These plots should be simply treated as trends, and not as absolute in terms of locations or magnitudes of the highest sensitivities. They represent how the sensitivity in a given length varies when *d* and *D* are varied. They do not necessarily represent the highest sensitivity that can be achieved for a given combination of *d* and *D*. For example, there may be some values of *d* and *D* at which a different line produces a higher sensitivity than that shown in these plots for that combination.

We noticed that the highest distance error sensitivity generally occurred at one of two configurations. In the first configuration, the detector is positioned closest to the source, and the stage is positioned closest to the detector. This configuration, corresponding to *d* = 200 mm and *D* = 300 mm, shall henceforth be referred to as the “near” configuration. The second configuration is the one in which the stage is positioned as far from the source as possible, and the detector is positioned as close to the stage as possible. This configuration, corresponding to *d* =1100 mm and *D* = 1200 mm, shall be called the “far” configuration. We discuss these configurations through a few examples as follows.

**Fig. 4:**
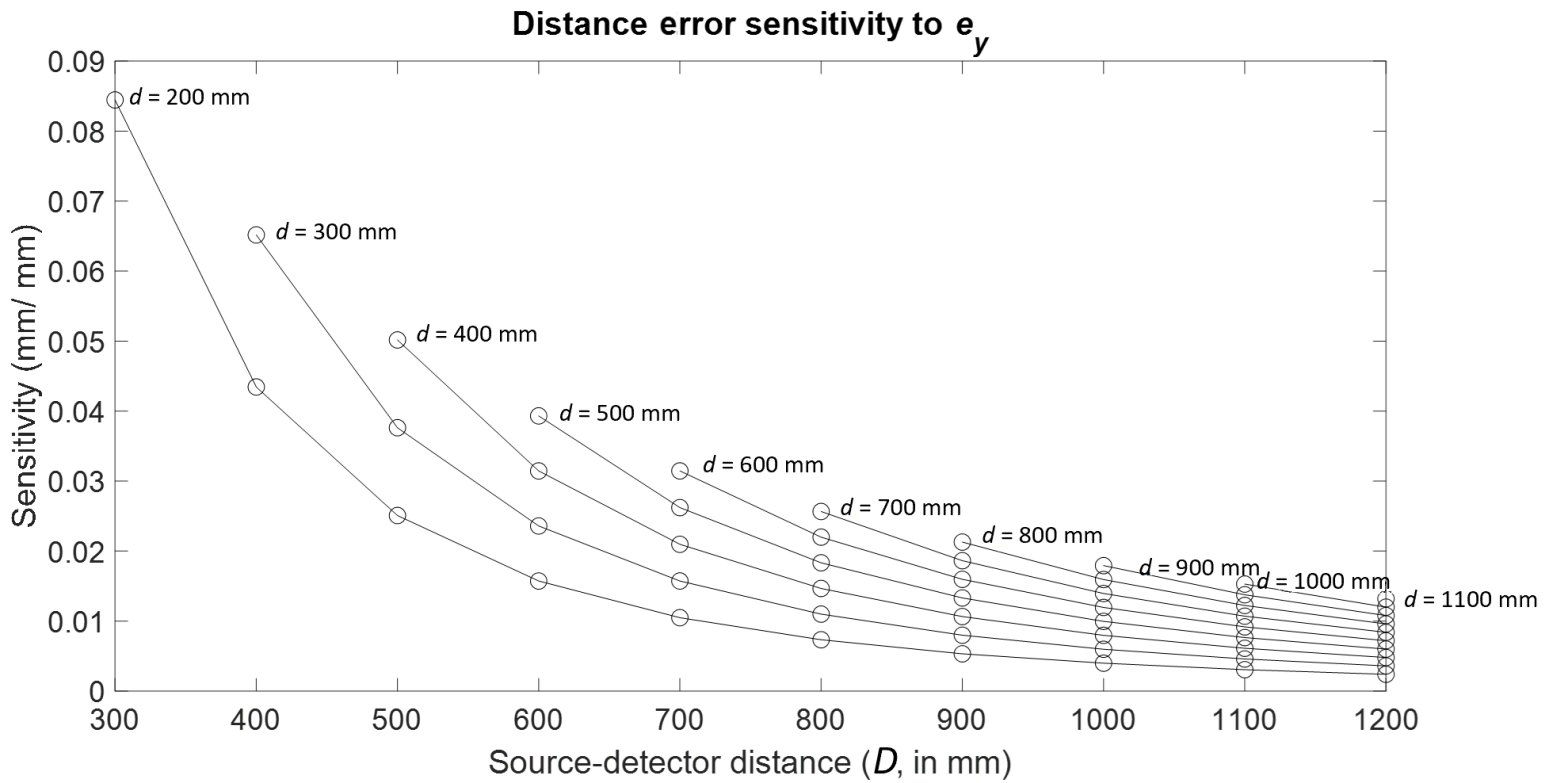
Distance error sensitivity to detector *Y* location error.

Consider the case of detector *Y* location error ($e_{y}$). We tracked one of the pairs of spheres identified in Fig. 3 for each of the 55 combinations of *d* and *D*. The results are plotted as a function of *d* and *D* in Fig. 4, where each curve in the plot represents the sensitivity for a fixed source-stage distance *d* and varying source-detector distances *D*. The sensitivities shown represent the ratio of the error (varying) in the calculated distance between a predetermined pair of spheres in the 125 sphere object and a given simulated error (constant) in the detector *Y* position. Clearly, the highest sensitivity to $e_{y}$ occurs at the near (*d* = 200 mm, *D* = 300 mm) configuration.

**Fig. 5:**
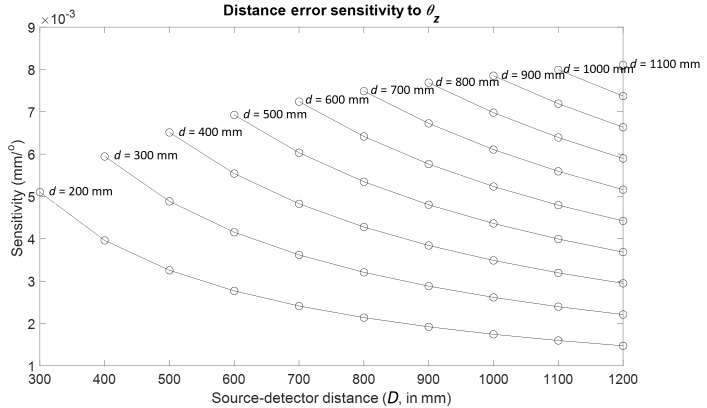
Distance error sensitivity to detector rotation about *Z*.

Consider next the case of detector rotation about the *Z* axis ($\theta_{z}$). We tracked one of the pairs of spheres identified in Fig. 3 for each of the 55 combinations of *d* and *D*. The results are plotted as a function of *d* and *D* in Fig. 5, where each curve in the plot represents the sensitivity for a fixed source-stage distance *d* and varying source-detector distances *D*. Clearly, the largest sensitivity occurs at the far (*d* = 1100 mm, *D* = 1200 mm) configuration. A summary of the results for all the detector errors can be found in Table 3.

#### 4.1.2 Rotation Stage Errors

**Fig. 6:**
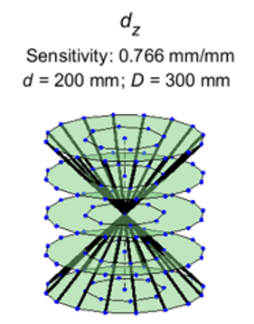
Lines of maximum sensitivity for the *Z* location error of the stage.

**Fig. 7:**
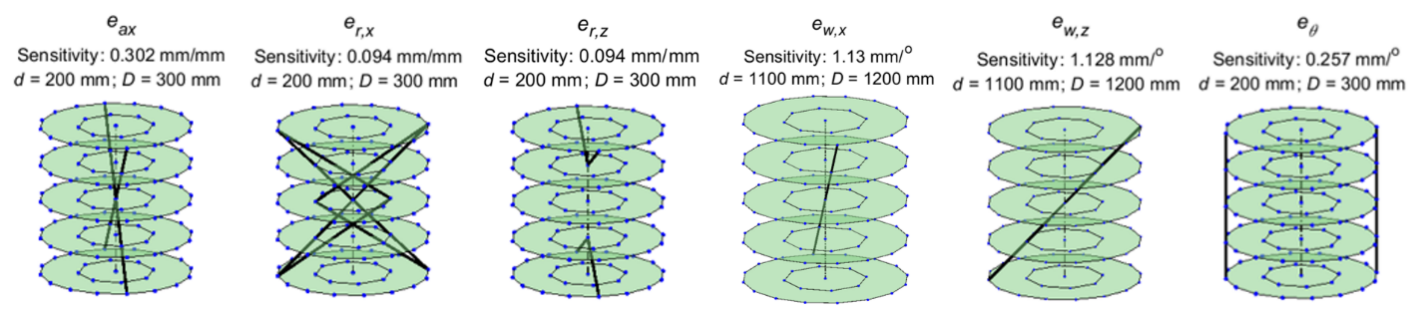
Lines of maximum sensitivity for first-order cosine components of stage error motions. From left to right: axial error, radial error along *X*, radial error along *Z*, wobble about *X*, wobble about *Z*, and scale errors in the encoder. These lines represent the optimal placement of a reference length that will produce the largest distance error for unit magnitude of each of these geometric error sources.

Here, we present the lines of maximum sensitivity for the stage errors. The sensitive lines for the *Z* location error of the stage are shown in Fig. 6. Figures 7 through 10 show similar illustrations of sensitive lines for the remainder of the stage errors, namely, the error motions of the stage. These errors are represented by sine and cosine components of orders 1 to 10. However, the magnitudes of sensitivities corresponding to the first four orders were found to be the most significant and are therefore shown here. Further, only the cosine components of the error sources are shown. The sensitive lines for the sine components were observed to have similar orientations but rotated about the rotation axis.

**Fig. 8:**
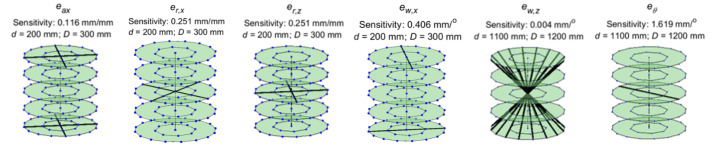
Lines of maximum sensitivity for second-order cosine components of stage error motions.

**Fig. 9:**
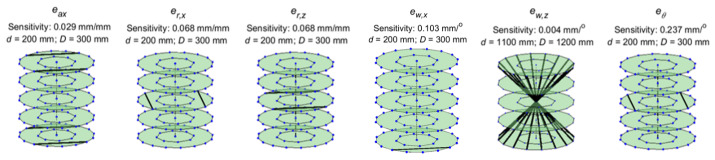
Lines of maximum sensitivity for third-order cosine components of stage error motions.

**Fig. 10:**
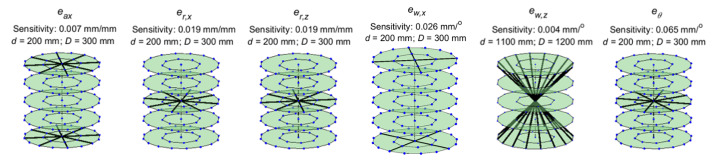
Lines of maximum sensitivity for fourth-order cosine components of stage error motions.

We next present examples of trends in sphere center-to-center distance errors for some rotation stage errors that have their highest sensitivities occurring in the near (*d* = 200 mm, *D* = 300 mm) and far (*d* = 1100 mm, *D* = 1200 mm) configurations.

**Fig. 11:**
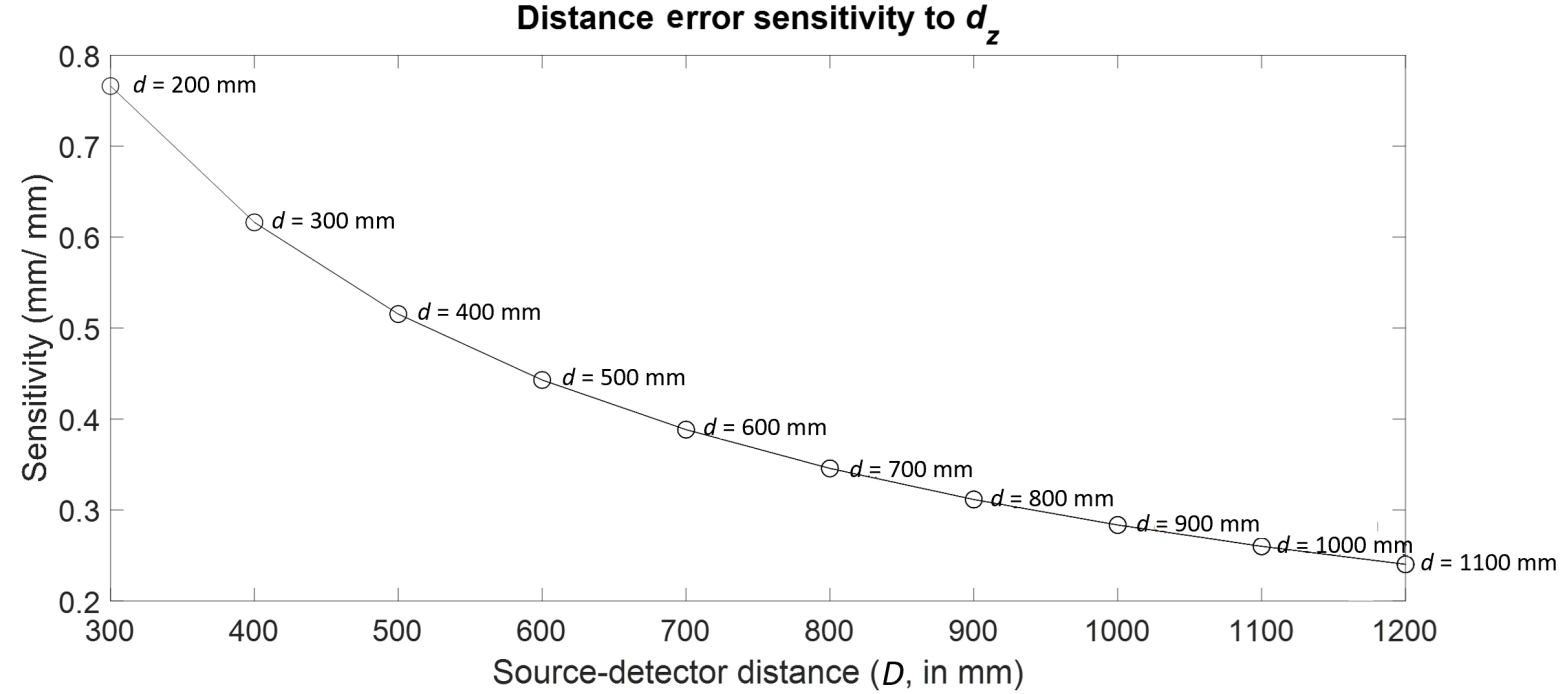
Distance error sensitivity to stage *Z* location error.

Consider the case of rotation stage *Z* location error. In Part II, we showed that this error source is sensitive to the long body diagonal in a given volume. We then tracked this length across different combinations of *d* and *D*. The results are shown in Fig. 11. Clearly, the largest sensitivity occurs at the near configuration, *i.e.*, with the stage located as close to the source as possible and the detector located as close to the stage as possible. The trends shown in Fig. 11 are similar to those shown in Fig. 4, except that the distances between the curves for different stage positions are so small that the curves appear to overlap.

**Fig. 12:**
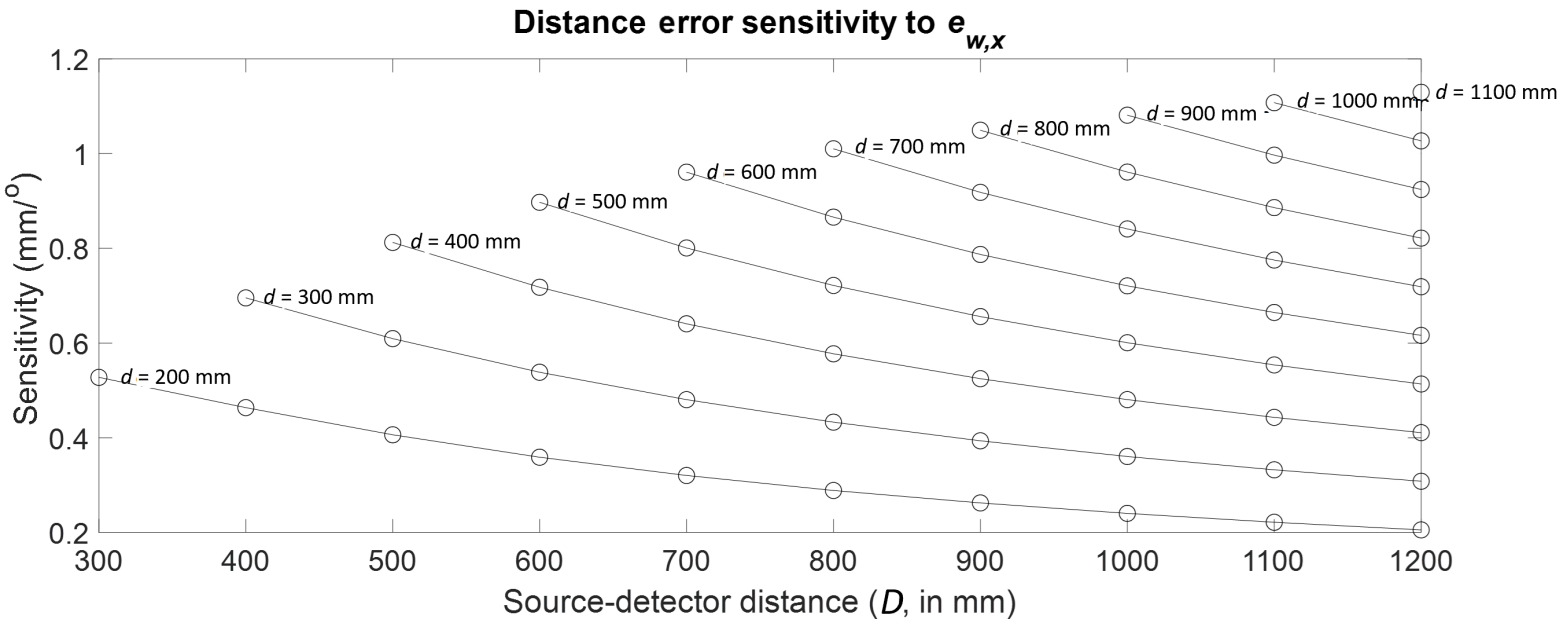
Distance error sensitivity to first-order cosine component of stage wobble along *X*.

Consider the case of the first-order cosine component of wobble along the *X* axis ($e_{w,x}$). The sensitive line is shown in Fig. 7. We tracked this line through each of the 55 combinations of *d* and *D*. The results are plotted as a function of *d* and *D* in Fig. 12, which clearly indicates that the largest sensitivity occurs at the far configuration. A summary of the results for all the stage errors can be found in Table 4.

#### 4.1.3 Observations and Results

Since the magnification for a given XCT measurement setup is determined by the ratio of *D* to *d*, consequently it is possible to achieve the same magnification by various combinations of *d* and *D*. For example, in Fig. 4, the configuration corresponding to *d* = 200 mm and *D* = 300 mm and the configuration corresponding to *d* = 400 mm and *D* = 600 mm both have the same magnification of value 1.5, but the sensitivities in the corresponding cases are significantly different. Therefore, it is clear that even when *d* and *D* are increased proportionately, *i.e.*, maintaining the same magnification, some combinations of stage and detector distances are more sensitive to, and therefore would more clearly reveal, certain error sources.

Trends similar to those shown in Figs. 4, 5, 11, and 12 were observed for all error sources, with the highest sensitivity occurring in the previously described near (*d* = 200 mm, D = 300 mm) or far (*d* = 1100 mm, *D* = 1200 mm) configuration. In both these configurations, the magnification is the lowest possible for a given stage position. This was found to be true of all error sources studied here.

The configurations that captured the highest distance error sensitivity for each error source are given in Table 3 and Table 4. The latter tabulates these configurations for the cosine components of the error motions of the stage, where *N* represents the order of the harmonic. The sine components of these errors are not shown here but exhibited similar trends.

Note that not all commercially available XCT systems have the benefit of being able to move the detector as freely as discussed here. In instances where the detector is fixed, the highest sensitivities possible for that case can be realized by simply moving the stage as close to the fixed detector as possible. This case is shown by the points corresponding to a single detector position, or a vertical line, in Figs. 4, 5, 11, and 12.

**Table 3:** Configuration capturing highest center-to-center distance error sensitivity for detector errors and stage *Z* location error.

Error Source	Configuration Showing Highest Sensitivity
$e_{x}$	Near
$e_{y}$	Near
$e_{z}$	Near
$\theta_{x}$	Near
$\theta_{y}$	Near
$\theta_{z}$	Far
$d_{z}$	Near

**Table 4:** Configuration capturing highest center-to-center distance error sensitivity for stage error motions.

Error Source	*N* = 1^a^	*N* = 2	*N* = 3	*N* = 4	*N* = 5	*N* = 6	*N* = 7	*N* = 8	*N* = 9	*N* = 10
$e_{ax}$	Near	Near	Near	Near	NS^b^	NS	NS	NS	NS	NS
$e_{r,x}$	Near	Near	Near	Near	Near	NS	NS	NS	NS	NS
$e_{r,z}$	Near	Near	Near	Near	Near	NS	NS	NS	NS	NS
$e_{w,x}$	Far	Near	Near	Near	Near	Near	NS	NS	NS	NS
$e_{w,z}$	Far	NS	NS	NS	NS	NS	NS	NS	NS	NS
$e_{\theta }$	Near	Far	Near	Near	Near	Near	NS	NS	NS	NS

^a^ *N*: Order of harmonic.

^b^ NS: Not significant (*i.e.*, sensitivities smaller than 0.001 mm/mm or 0.001 mm/°), where all configurations show similar sensitivity and negligible magnitude.

### 4.2 Sphere Form Errors

#### 4.2.1 Detector Errors

For most of the error sources in this study, sensitivities for sphere form errors also showed trends similar to those of center-to-center distance errors in that maximum sensitivity was found at the near (*d* = 200 mm, *D* = 300 mm) and far (*d* = 1100 mm, *D* = 1200 mm) configurations previously described. In some cases, the peak sensitivity occurred at other configurations. However, in these rare cases, the sensitivity differed negligibly across the different configurations.

Further, for almost all error sources and for almost all *d* and *D* values, the spheres furthest away from the axis of rotation (*i.e.*, spheres in the outer ring) produced the highest form error. Even in cases where the spheres that produced the highest form error were located in other positions (described by “Other” in Table 6), their form error often differed from those of the outer ring of spheres by very little. Here, we present examples of trends in form error sensitivities where peak sensitivities were found at the near (*d* = 200 mm, *D* = 300 mm) and far (*d* = 1100 mm, *D* = 1200 mm) configurations. Note that the actual magnitude of the form error is somewhat influenced by the implementation of the SPRT method when generating the form error point cloud. We noticed differences in form error of about 10% between different implementations, especially between algorithms that retain and algorithms that discard data near the poles of the sphere.

**Fig. 13:**
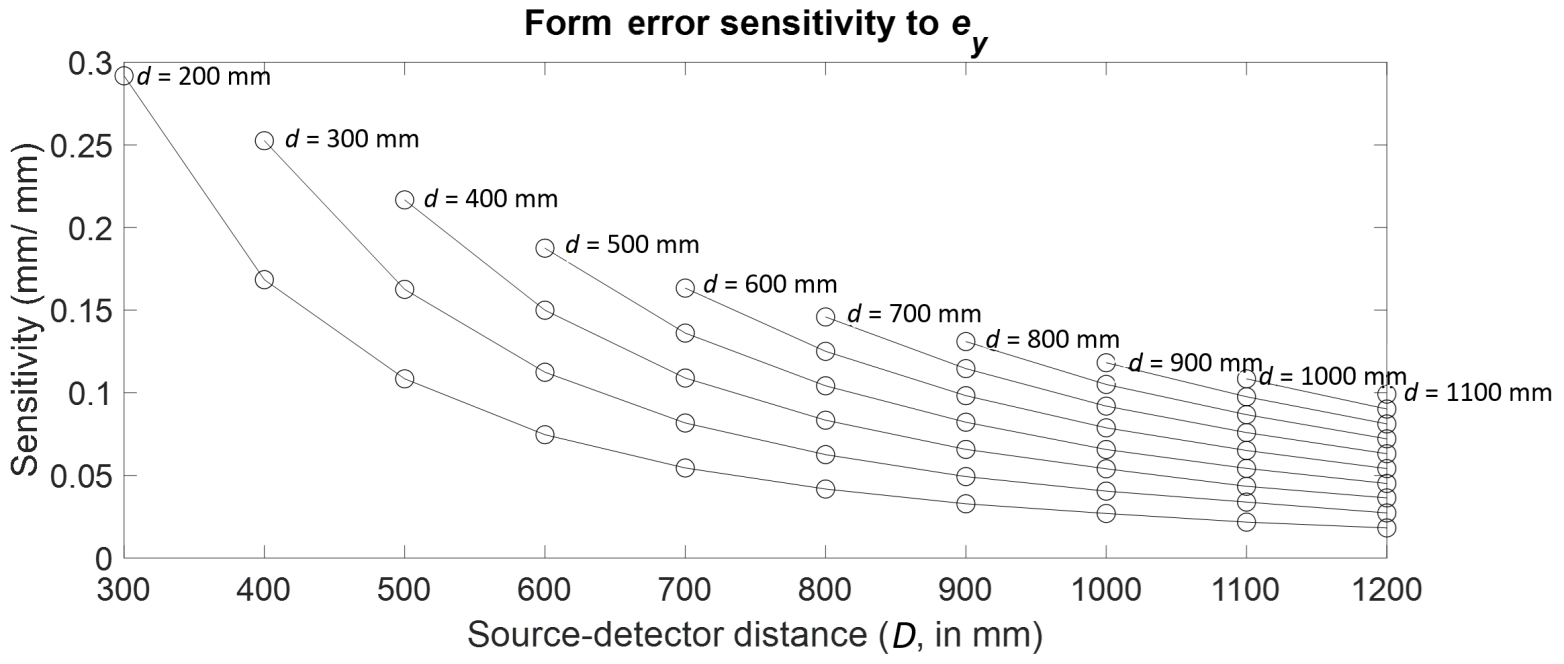
Form error sensitivity to detector *Y* location error.

Consider the case of detector *Y* location error ($e_{y}$). One sphere in the outer ring of spheres was tracked through the 55 combinations of stage and detector positions, and the form error sensitivities are plotted as a function of *d* and *D* in Fig. 13. Clearly, the largest sensitivity occurs at the near (*d* = 200 mm, *D* = 300 mm) configuration.

**Fig. 14:**
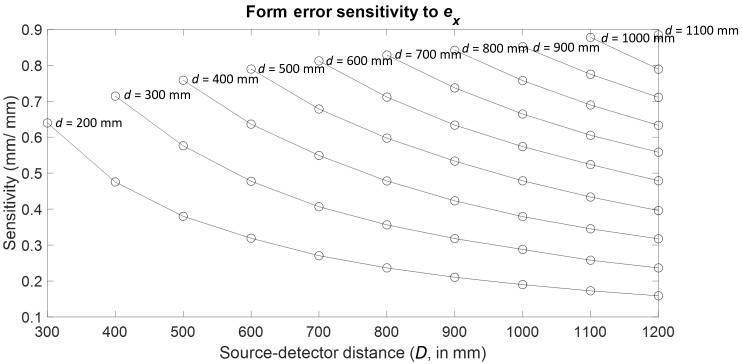
Form error sensitivity to detector *X* location error.

Consider the case of detector *X* location error ($e_{x}$). As before, a sphere in the outer ring was tracked through the 55 combinations of stage and detector positions, and the resulting form error sensitivities are plotted as a function of *d* and *D* in Fig. 14. Clearly, the largest sensitivity occurs at the far (*d* = 1100 mm, *D* = 1200 mm) configuration. A summary of the results for all the detector errors can be found in Table 5.

#### 4.2.2 Rotation Stage Errors

#### The form error sensitivities for stage errors showed trends similar to those of detector errors in terms of location of the spheres that produced the highest sensitivity, as well as the tendency to peak at the near (*d* = 200 mm, *D* = 300 mm) or far (*d* = 1100 mm, *D* = 1200 mm) configurations. We present some examples of stage errors where peak sensitivities occurred at these near and far configurations.

**Fig. 15:**
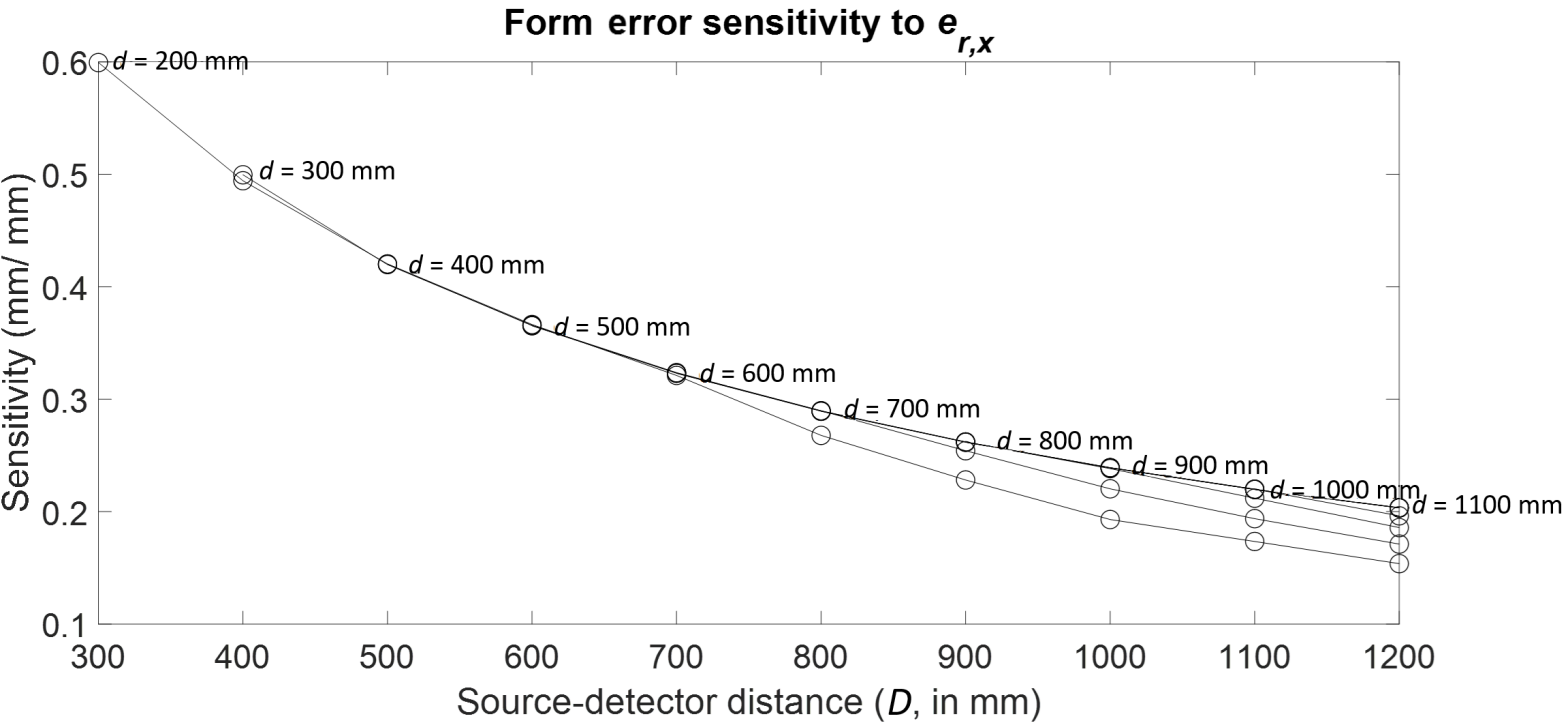
Form error sensitivity to first-order cosine component of stage radial error along *X*.

Consider the case of the first-order cosine component of the stage radial error in *X* ($e_{r,x}$). One sphere in the outer ring of spheres was tracked through the 55 combinations of stage and detector positions, and the results are plotted as a function of *d* and *D* in Fig. 15. Clearly, the largest sensitivity occurs at the near configuration. Similar to Fig. 11, the distances between the curves in Fig. 15 corresponding to different stage positions are so small that the curves appear to overlap to a certain extent.

**Fig. 16:**
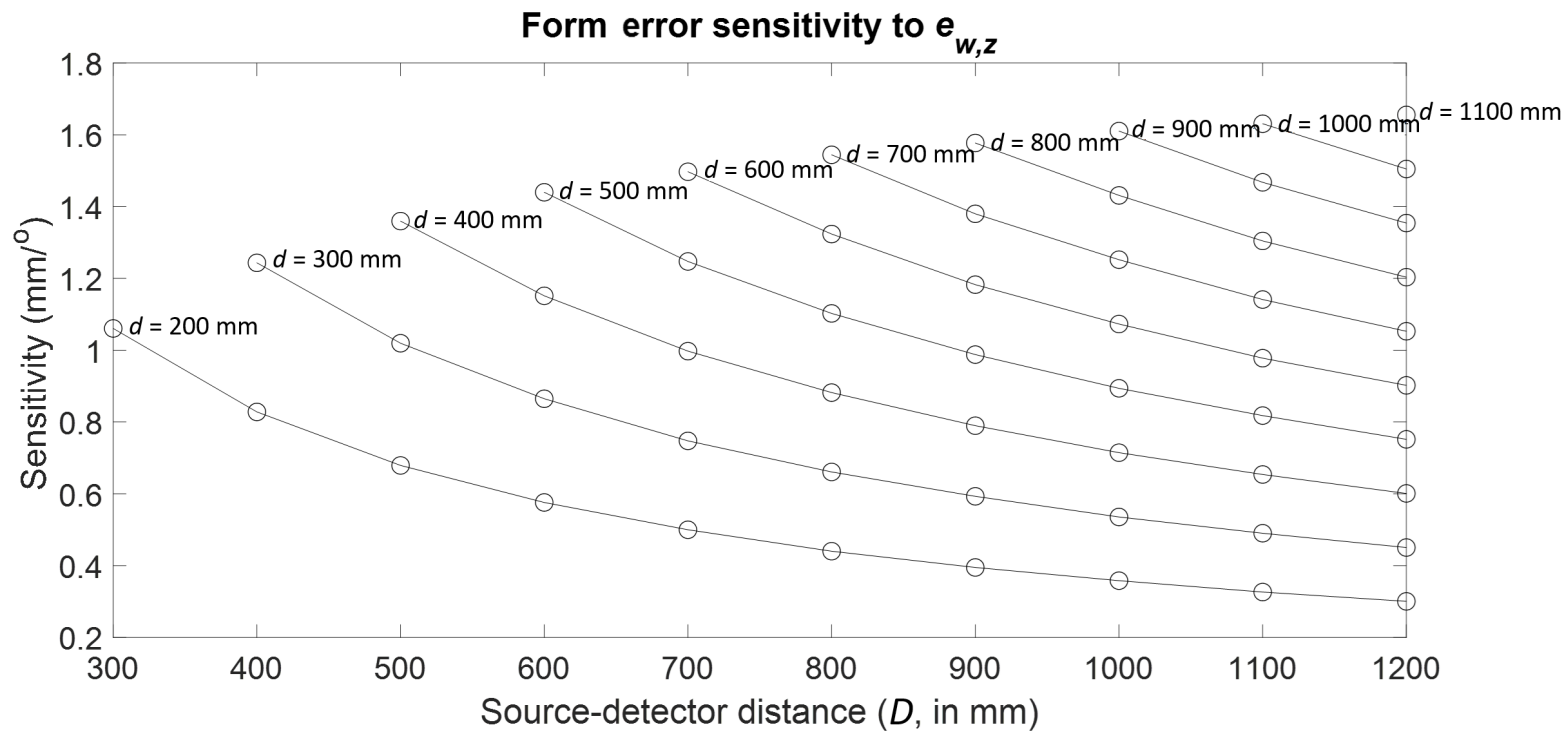
Form error sensitivity to first-order cosine component of stage wobble along *Z*.

Consider the case of the first-order cosine component of the stage wobble in *Z* ($e_{w,z}$). A sphere in the outer ring of spheres was tracked through the 55 combinations of stage and detector positions, and the results are plotted as a function of *d* and *D* in Fig. 16. Clearly, the largest sensitivity occurs at the far configuration. A summary of the results for all the stage errors can be found in Table 6.

#### 4.2.3 Observations and Results

Table 5 and Table 6 show the configurations that captured the highest form error sensitivities for each of the error sources. Table 6 contains the configurations that best captured the cosine components of the error motions of the stage, where *N* denotes the order of the harmonic. The sine components of these errors are not reported here but exhibited similar trends.

**Table 5:** Configuration capturing highest form error sensitivity for detector errors and stage *Z* location error.

Error Source	Configuration Showing Highest Sensitivity
$e_{x}$	Far
$e_{y}$	Near
$e_{z}$	Near
$\theta_{x}$	NSa
$\theta_{y}$	Near
$\theta_{z}$	Far
$d_{z}$	NS

a NS: Not significant, where all configurations showed similar sensitivity and negligible magnitude.

**Table 6:** Configuration capturing highest form error sensitivity for stage error motions.

Error Source	*N* = 1^a^	*N* = 2	*N* = 3	*N* = 4	*N* = 5	*N* = 6	*N* = 7	*N* = 8	*N* = 9	*N* = 10
$e_{ax}$	Near	Far	Near	Far	Other b	Far	Other b	Far	Far	Far
$e_{r,x}$	Near	Near	Far	Near	Near	Near	Far	Other b	Far	Other b
$e_{r,z}$	Near	Near	Near	Near	Near	Near	Near	Near	Near	Near
$e_{w,x}$	Far	Far	Far	Far	Far	Far	Far	Far	Far	Far
$e_{w,z}$	Far	Far	Far	Far	Far	Far	Far	Far	Far	Far
$e_{\theta }$	Far	Far	Far	Far	Far	Far	Far	Far	Far	Far

a *N*: Order of harmonic (in this study, we considered the first ten orders for each of the sine and cosine components of the stage rotation errors).

b “Other” means that the maximum sensitivity occurred at a position that was neither the “near” configuration nor the “far” configuration. The choice of algorithm in the implementation of the SPRT influences the location of maximum sensitivity.

For all error sources, unless the sphere form errors were negligible for all spheres, one of the two following cases was observed:

(1) Spheres with the highest form error were those that were radially the furthest from the axis of rotation, i.e., independent of their height in the measurement volume.
(2) Spheres with the highest form error were those that were radially furthest from the axis of rotation as well as furthest from the Y = 0 plane (i.e., the horizontal plane located at the middle of the measuring volume).

## 5 Conclusions

This work has reported, by means of simulation studies, the positions of stage and detector that result in high center-to-center distance error and sphere form error sensitivities for all geometric errors associated with the stage and detector in a given XCT instrument and identified the measurement lines and spheres within the corresponding measurement volume that produce these highest distance errors and form errors, respectively.

The key contributions of Part III in this series are as follows:

• In Parts I and II, we identified the location of a sphere in a given measurement volume that resulted in the largest form error for a given geometry error source. Here, we tracked that sphere for different combinations of *d* and *D* and noted the stage and detector positions where the largest sensitivity occurred.
• In Parts I and II, we identified the pair of spheres that produced the largest distance error for a given error source. Here, we tracked that pair of spheres for different combinations of *d* and *D* and noted the stage and detector positions for which this largest error occurred.
• We abstracted the information from these simulation studies, resulting in the following main observations:
  o We identified two combinations of rotation stage and detector locations that result in large sensitivities; we refer to these combinations as the near position and the far position.
  o In the near position, the rotation stage is closest to the source, and the detector is closest to the rotation stage, *i.e.*, *d* = 200 mm and *D* = 300 mm in our simulations. In, the far position, the detector is farthest from the source, and the rotation stage is closest to the detector, *i.e.*, *d* = 1100 mm and *D* = 1200 mm in our simulations. Note that both the near and far positions are low-magnification positions, *i.e.*, with the detector as close as possible to the rotation stage.

We observed that, for the purposes of performance testing, specifying magnification is not sufficient. The positions of the detector and rotation stage (or *d* and *D* values) need to be explicitly stated.
